# Keratinocyte Antiviral Response to Poly(dA:dT) Stimulation and Papillomavirus Infection in a Canine Model of X-Linked Severe Combined Immunodeficiency

**DOI:** 10.1371/journal.pone.0102033

**Published:** 2014-07-15

**Authors:** Jennifer A. Luff, Hang Yuan, Douglas Kennedy, Richard Schlegel, Peter Felsburg, Peter F. Moore

**Affiliations:** 1 Department of Veterinary Pathology, Microbiology, and Immunology, School of Veterinary Medicine, University of California Davis, Davis, California, United States of America; 2 Department of Pathology, Georgetown University Medical School, Washington, D.C., United States of America; 3 Department of Immunology, School of Veterinary Medicine, University of Pennsylvania, Philadelphia, Pennsylvania, United States of America; University of California, San Francisco, United States of America

## Abstract

X-linked severe combined immunodeficiency (XSCID) is caused by a genetic mutation within the common gamma chain (γ_c_), an essential component of the cytokine receptors for interleukin (IL)-2, IL-4, IL-7, IL-9, IL-15, and IL-21. XSCID patients are most commonly treated with bone marrow transplants (BMT) to restore systemic immune function. However, BMT-XSCID humans and dogs remain at an increased risk for development of cutaneous papillomavirus (PV) infections and their associated neoplasms, most typically cutaneous papillomas. Since basal keratinocytes are the target cell for the initial PV infection, we wanted to determine if canine XSCID keratinocytes have a diminished antiviral cytokine response to poly(dA:dT) and canine papillomavirus-2 (CPV-2) upon initial infection. We performed quantitative RT-PCR for antiviral cytokines and downstream interferon stimulated genes (ISG) on poly(dA:dT) stimulated and CPV-2 infected monolayer keratinocyte cultures derived from XSCID and normal control dogs. We found that XSCID keratinocytes responded similarly to poly(dA:dT) as normal keratinocytes by upregulating antiviral cytokines and ISGs. CPV-2 infection of both XSCID and normal keratinocytes did not result in upregulation of antiviral cytokines or ISGs at 2, 4, or 6 days post infection. These data suggest that the antiviral response to initial PV infection of basal keratinocytes is similar between XSCID and normal patients, and is not the likely source for the remaining immunodeficiency in XSCID patients.

## Introduction

Severe combined immunodeficiency (SCID) represents a diverse group of genetic diseases that results in either absent or non-functional B and T lymphocytes. These patients are highly susceptible to infectious diseases and death usually occurs by one to two years of age. The most common form of SCID in humans is X-linked SCID (XSCID). It is caused by a genetic mutation within the common gamma chain (γ_c_), an essential component of the cytokine receptors for IL-2, IL-4, IL-7, IL-9, IL-15, and IL-21 [1]. Several other genetic mutations, including mutations within Jak3 (an essential part of the γ_c_ signaling pathway), can also result in SCID. Patients with SCID are most commonly treated with bone marrow transplants (BMT) in order to restore immune function and dramatically increase patient survival. However, some of these patients remain at an increased risk for development of certain infections, including infections with papillomaviruses (PV) that can result in formation of debilitating papillomas [2], [3], [4].

In a recent retrospective study on the long-term outcome of 90 BMT SCID patients, 26% (23/90) had developed cutaneous human papillomavirus (HPV) disease [4]. The vast majority of these patients had molecular defects within γ_c_ (11/23) or Jak3 (8/23). Two other long-term follow up studies on BMT SCID patients had similar results. In both of these studies, the SCID patients that developed chronic cutaneous HPV disease (9/41 and 9/76) all had molecular defects within either γ_c_ or Jak3 [2], [3]. Patients with SCID due to other molecular defects did not show this increased risk for HPV infections.

A similar increase in risk for cutaneous PV infections and development of associated papillomas has been observed in a research colony of dogs with a genetic mutation within γ_c_ [5]. Their similar clinical and immunological phenotype to human patients has made them a successful animal model to study XSCID [6], [7]. These XSCID dogs are treated with BMTs or gene therapy, which has resulted in full immunologic reconstitution of both B and T lymphocytes [8], [9], [10], [11]. However, despite their apparently normal systemic immune function after treatment, many of these dogs, 71% (17/24), develop severe cutaneous PV disease; specifically, they are infected with canine papillomavirus-2 (CPV-2), a cutaneous pathogen that manifests as non-regressing papillomas that progress to metastatic cancer [5], [12]. Immunocompetent littermate dogs within the same research colony have never developed papillomas, suggesting that although the systemic immune system is restored after BMT, there is still a persistent immune deficiency within these XSCID animals that likely reflects what is occurring in human XSCID patients.

The keratinocyte is the target cell for these cutaneous PV infections. Even after successful BMTs, the keratinocyte likely remains deficient in γ_c_ signaling, as they are not replaced after bone marrow transplantation. They represent a possible source for these patients' continued immunodeficiency. While the role of the γ_c_ –dependent cytokine receptors in lymphocyte function has been well-characterized, there is relatively little know about their function(s), if any, within keratinocytes. One of the γ_c_ -dependent cytokines, IL-15, has been recently recognized as an important antiviral cytokine. It can mediate the release of type 1 interferons (IFN) and other antiviral cytokines as well as play an essential role in protective immunity to certain viral infections [13], [14], [15]. We wanted to determine if lack of γ_c_-dependent cytokine signaling in XSCID keratinocytes would result in a diminished antiviral immune response to the initial cutaneous PV infection, which occurs in basal keratinocytes. We first examined the response to a synthetic analog of viral dsDNA [poly(dA:dT)] and then to CPV-2, looking specifically at cytokines and interferon stimulated genes (ISG) that our laboratory has previously shown to be upregulated in normal keratinocytes in response to poly(dA:dT) [16]. These included several of the cytosolic nucleic acid sensing pattern recognition receptors (PRRs): melanoma differentiation associated gene 5 (MDA5), retinoic acid-inducible gene I (RIG-I), and IFI16 (interferon inducible gene 16) [17]. We chose these PRRs since they are important for viral recognition and can be upregulated after stimulation with PRR ligands [18]. We also looked at the ISG interferon-induced protein with tetratricopeptide repeats 1 (IFIT1) [19]. In our previous study, we were unable to elicit an immune response to CPV-2 in normal keratinocytes [16]. We still, however, wanted to investigate the response of CPV-2 infection in XSCID kerationcytes based upon the clinical observations of the seemingly increased susceptibility to PV infections in these XSCID patients.

## Materials and Methods

### Ethics statement

This study was carried out in strict accordance with the recommendations in the Guide for the Care and Use of Laboratory Animals of the National Institutes of Health. The protocol for XSCID dog studies was approved by the Institutional Animal Care and Use Committee at the University of Pennsylvania (IACUC # 804580). The samples of skin from normal dogs were obtained from the discarded fresh materials submitted for biopsy evaluation under consent of the owner. The protocol was approved by the Institutional Animal Care and Use Committee at the University of California, Davis (IACUC # 16686).

### 2.1 Isolation and culture of normal and XSCID keratinocytes

Normal canine skin was obtained from discarded fresh biopsy specimens or unrestricted euthanized dogs submitted to the pathology department at the University of California, Davis Veterinary Medical Teaching Hospital under review of the Clinical Trial Review Board for tissue banking samples. Three XSCID dogs (listed in Table 1) were used to isolate and establish primary XSCID canine keratinocyte cultures under the ethical guidelines of the University of Pennsylvania's Institutional Animal Care and Use Committee. These XSCID dogs all had a 4-bp deletion within exon 1 of γ_c_ and were derived from a breeding colony established from a single carrier female [6], [7]. Two of the dogs received BMTs based upon previously established methods [3], [8], [9]. One of the dogs received gene therapy for γ_c_ based upon previously established methods [11]. All of the dogs were humanely euthanized due to formation of debilitating footpad papillomas; one dog's (R1752) papilloma progressed to squamous cell carcinoma. Normal skin samples were obtained at the time of euthanasia. Isolation and culture of primary canine keratinocytes was performed as previously described [16], [20], [21]

**Table 1:** Treatment type, date of treatment, and course of papillomavirus infection in XSCID dogs from this study.

Dog	Treatment	Date of therapy (mo/day/yr)	Date of First Papilloma (mo/yr)	Date of SCC (mo/yr)	Status
R1752	BMT	6/7/05	4/08	10/10	Euthanized (10/10)
R2034	Gene Tx	10/8/08	5/09	NA*	Euthanized (5/11)
R2155	BMT	12/15/10	5/18/11	NA	Euthanized (1/12)

* No SCC; SCC =  squamous cell carcinoma.

### 2.2 Ligand stimulation and CPV-2 infection

Propagation, purification, and quantification of infectious CPV-2 viral particles were performed as previously described using canine papilloma-derived particles implanted into SCID mice [16]. The stimulation assay and CPV-2 infection was performed as described previously [16]. Briefly, 3.0×10^5^ viable keratinocytes (between passage 5–10) from normal and XSCID dogs were seeded into multiple wells of 6-well tissue culture plates (Fischer Scientific, BD Falcon, Hanover Park, IL, USA) and incubated at 34°C and 5% CO_2_ for 24 hours. After 24 hours, the cells were incubated in medium alone, medium containing 0.6 ug/ml poly(dA:dT)/Lyovec (Invivogen, San Diego, CA, USA) or medium containing 200 CPV-2 viral particles per cell. The cells were incubated for an additional 2, 4, or 6 days before RNA extraction. All reactions were performed in triplicate and repeated in three independent experiments. Each independent experiment used primary keratinocyte cultures derived from a different normal control dog (n = 3) and one of two different primary keratinocyte cultures derived from XSCID dogs (n = 2). We were unable to establish primary keratinocyte culture from one of the three XSCID dogs, (R2034).

Since PVs are non-cytolytic viruses and do not produce virions in monolayer cultures, confirmation of *in vitro* PV infection is most commonly based upon the presence of mRNA spliced transcripts of the viral early genes [22]. In this study, we used RT-PCR to detect the E1∧E2 spliced transcripts to confirm PV infection of the keratinocytes, as described previously [16].

### 2.3 Total RNA extraction and cDNA preparation

Total RNA was extracted using a RNA extraction kit (Qiagen RNeasy mini kit, Qiagen, Valencia, CA, USA) following manufactures recommended protocols. Resulting RNA was DNase treated and complementary DNA (cDNA) generated using a reverse transcription kit (Quantitec RT kit, Qiagen). A no reverse transcriptase (RT-) sample was used to control for genomic DNA contamination.

### 2.4 Primer design, validation, and conventional PCR

Primers for IFIT1, γ_c_, IL-2Rα, IL-4Rα, IL-7Rα, IL-9Rα, IL-15Rα, and IL-21Rα were designed based upon the reference mRNA sequence listed in the NCBI database using commercially available primer design software (Oligo Primer Analysis Software, Molecular Biology Insights, Inc., West Cascade, CO, USA). The primer sequences and their corresponding reference mRNA sequences are listed in Table 2. Primer sets for GAPDH, E1∧E2, IFI16, IFN-κ, RIG-I, and MDA5 have been previously published [16]. Conventional PCR was performed on an Applied Biosystems GeneAmp PCR system 2700 thermocycler (Foster City, CA, USA) using previously described methods [16]. The annealing temperature used for each primer pair is listed in Table 2. Conventional RT-PCR for the γ_c_ -dependent cytokine receptors was performed as follows: 95°C for 10 minutes followed by 35 cycles of 1) 1 minute at 95°C 2) 1 minute at Ta°C, and 3) 1 minute at 72°C and concluded with 10 minutes at 72°C. The PCR products were size separated and visualized using an eGene HAD-GT12 capillary electrophoresis analyzer (renamed QIAxcel, Qiagen, Valencia, CA, USA).

**Table 2:** Primer sets, product size, and GenBank accession numbers for RT-PCR.

Target gene	GenBank accession no.	Primer sequence forward and reverse (5'—3')	Product size (bp)	Annealing Temp (°C)
IL-2Rα	NM_001003211.1	CAGCCCCCGCTCAAGTGCAT	207	55
		GACGCAGCTCGCCACTGCTA		
IL-4Rα	XM_547077.2	TGCACGAGCCCAGCTGCTTC	307	65
		ACTGTGAGGTTGCCGGGGGT		
IL-7Rα	XM_850315.1	ACTCCAGAGATCACAGGGCGGA	342	65
		CTGCAAGCCCCCTCCAAGCC		
IL-9Rα	XM_005621780.1	GGGCAGGAGCAGCACTATGA	154	65
		GCAGCAGAAAGACGGGCACA		
IL-15Rα	XM_005617203.1	TGCCTCCCAGGAGACGCCAG	287	65
		GCGCCTTCGCTTCAGCCTCT		
IL-21Rα	XM_844902.1	GCTCCAGGGAGCCTGGGGTT	298	65
		AAAGCTGCCGCACTCCTGGG		
γ_c_	NM_001003201.1	TGGGACCGCAGCTGGACTGA	288	55
		TCGGGGGATCGACCGTTCCAG		
γ_c_ *	NM_001003201.1	TACACCCAGGGAACGAAGAGC	110/114	65
		GCATGGGGACCGTGGA		
IFIT1	XM_843271.2	TGGCAGTGCAGAGGTCAGGATAG	119	57
		GCCAGGTGTATATAAGCAATGTCAA		

*Primer pair used to detect 4-bp deletion in XSCID keratinocytes.

### 2.5 Quantitative RT-PCR

Real time RT-PCR for IFI16, IFN-κ, RIG-I, MDA5, and IFIT1 was performed using SYBR green detection following standard protocols as previously described [16]. Product specificity was monitored by a final melting curve analysis. Real time RT-PCR for β-actin, IFN-β, IL-6, IL-10, IL-12A, and TNF-α was performed using the validated Taqman® Gene Expression Assay system (Applied Biosystems, Foster City, CA) with the following primer and probe sets as previously described: β-actin (Cf03023880_g1), IFN-β (Cf03644503_s1), IL-6 (Cf02624282_m1), IL-10 (Cf02624265_m1), IL-12A (Cf02690011_m1), and TNF-α (Cf02628237_m1). All PCR reactions were performed on a 7300 Real Time PCR System (Applied Biosystems). The expression of individual genes was normalized to expression of GAPDH (SYBR green detection method) or β-actin (Taqman detection method) and graphed as the relative fold change between the experimental (stimulated/infected) and calibrator (un-stimulated sample from same time point) based upon the 2-ΔΔCq method.

## Results

### 3.1 Confirmation of γ_c_ mutation in XSCID primary keratinocyte cultures

**Figure 1:**
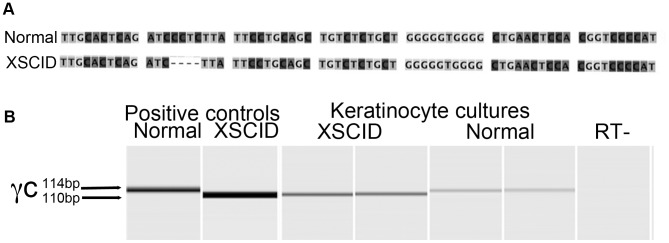
Confirmation of γ_c_-deficient primary keratinocyte cultures from bone marrow transplanted XSCID dogs. **A** The genetic mutation in XSCID dogs is a 4 base pair deletion in exon 1 of γ_c_. **B** RT-PCR was performed in duplicate on cDNA isolated from primary keratinocyte cultures using primer pairs that span the 4 base pair deletion in γ_c_. Results are shown from one experiment performed on one normal dog and one XSCID dog. Similar results were obtained from two other experiments using keratinocytes derived from a second XSCID dog and two normal control dogs. The PCR product size from normal dogs is 114 bps and from XSCID dogs is 110 bps. Positive controls included genomic DNA from normal canine skin and splenic genomic DNA from non-bone marrow transplanted XSCID dog.

Primary keratinocyte cultures used in this study were established from 3 normal dogs and 2 BMT-XSCID dogs (attempt at primary keratinocyte cultures from the third XSCID dog that underwent gene therapy were unsuccessful). RT-PCR was performed using primers that span the known 4-bp deletion in exon 1 of γ_c_ in XSCID dogs [23]. This was performed in order to confirm that the established XSCID keratinocyte cultures maintained defective γ_c_ mRNA and thus defective protein. The 4-bp deletion in the mRNA results in a premature stop codon and a truncated, dysfunctional γ_c_ protein [23]. The size of the PCR product from the primary keratinocyte cultures derived from XSCID dogs was 110 bp while the size of the PCR product from normal control dogs was 114 bp (Figure 1).

### 3.2 Baseline γ_c_ -dependent cytokine receptor mRNA expression in canine keratinocyte cultures

**Figure 2:**
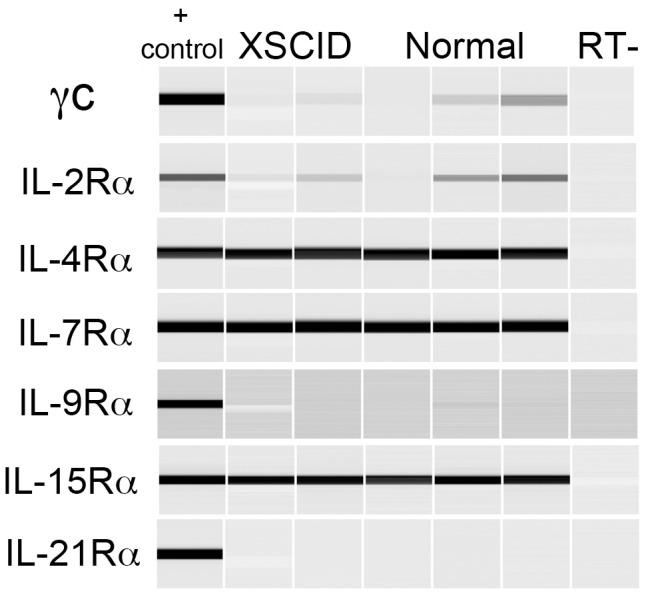
Expression of γ_c_ -dependent cytokine receptors in XSCID and normal keratinocyte cultures. RT-PCR for γ_c_ and alpha subunits was performed on monolayer keratinocyte cultures 24 hours after seeding. Results shown are from cultures derived from two XSCID dogs and three normal control dogs. Positive control included cDNA from either canine histiocytic or lymphocytic cell lines.

The γ_c_ -dependent cytokine receptors are type 1 cytokine receptors composed of the γ_c_ - subunit and a ligand-specific α-subunit. Both the IL-2 and IL-15 receptors have an additional shared β-subunit [1]. The primers successfully amplified the target cDNA sequence for each of the canine γ_c_ -dependent cytokine receptor α-chain subunits.RT-PCR for the γ_c_ dependent cytokine receptor α- and γ_c_ -subunits was performed on canine monolayer cultures derived from 3 different normal control dogs and 2 XSCID dogs. As shown in Figure 2, both normal and XSCID cultured keratinocytes expressed abundant mRNA for IL-4Rα, IL-7Rα, and IL-15Rα, low/variable expression for γ_c_ and IL-2Rα and no expression of IL-9Rα or IL-21Rα.

### 3.3 Kinetics of cytokine and ISG mRNA expression in normal and XSCID keratinocytes stimulated with synthetic dsDNA

**Figure 3:**
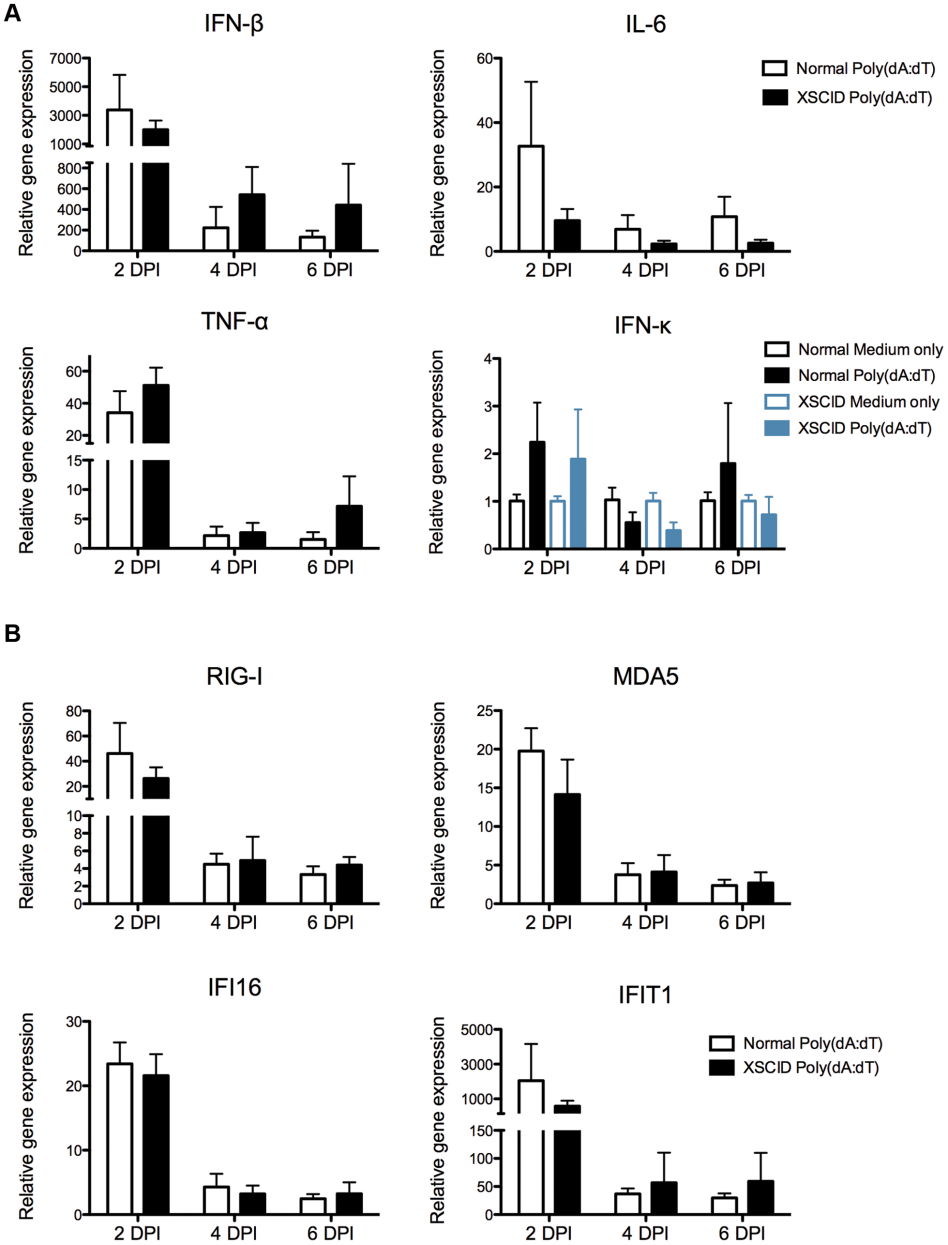
Kinetics of cytokine and interferon stimulated gene expression in XSCID canine keratinocytes stimulated with poly(dA:dT). **A and B** Keratinocytes were seeded into multiple wells and cultured as a monolayer for 24(dA:dT) or medium alone. RNA was extracted after 2, 4, and 6 days post-stimulation. Cytokine (**A**) and interferon stimulated gene (**B**) expression was determined by quantitative RT-PCR. Resulting Cq values were normalized to a reference gene and calibrated to mRNA expression in unstimulated keratinocytes (ΔΔCq). Results are expressed as mean +/− SD of three replicate experiments performed in triplicate. Each experiment used keratinocytes derived from a different normal control dog (n = 3) and one of two different XSCID dogs (n = 2).

Primary keratinocyte cultures from normal and XSCID dogs were stimulated with poly(dA:dT) for 2, 4, and 6 days to assess the mRNA expression of pro-inflammatory cytokines and ISGs using quantitative RT-PCR. Both normal and XSCID keratinocytes upregulated mRNA for IFN-β, IL-6, and TNF-α in response to poly(dA:dT) (Figure 3A) and this response was similar between normal and XSCID keratinocytes. The wide standard deviation shown in Figure 3 reflects the variability observed between individual dogs from each of the independent experiments. The greatest mRNA expression was seen at 48 hours. The mRNA expression of IFN-κ was not significantly upregulated at any time-point for either normal or XSCID dogs.

The mRNA expression of IFI16, MDA5, RIG-I, and IFIT1 in both normal and XSCID keratinocytes was similarly upregulated after stimulation with poly(dA:dT) (Figure 3B). Highest expression was observed at 48 hours for both XSCID and normal keratinocytes. IFIT1 revealed the greatest fold change between unstimulated and stimulated keratinocytes and results were similar between normal and XSCID keratinocytes.

### 3.4 Kinetics of cytokine and ISG mRNA expression in normal and XSCID keratinocytes infected with CPV-2

**Figure 4:**
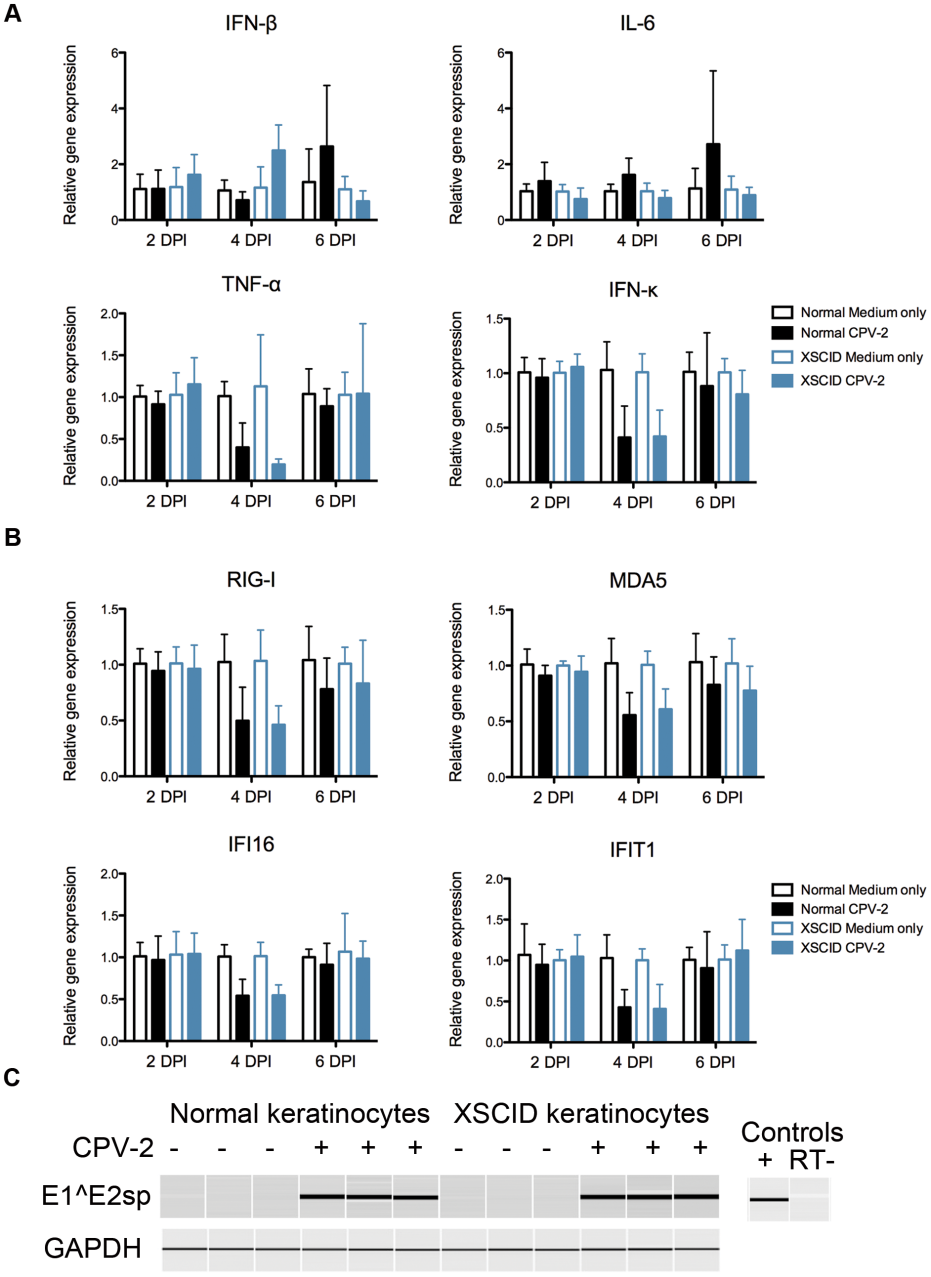
Kinetics of cytokine and interferon stimulated gene expression in XSCID canine keratinocytes infected with CPV-2. **A and B** Keratinocytes were seeded into multiple wells and cultured as a monolayer for 24-2 (CPV-2) (200 viral particle per cell) or medium alone. RNA was extracted after 2, 4, and 6 days post-infection (DPI). Cytokine (**A**) and interferon stimulated gene (**B**) expression was determined by quantitative RT-PCR. Resulting Cq values were normalized to a reference gene and calibrated to mRNA expression in unstimulated keratinocytes (ΔΔCq). Results are expressed as mean +/− SD of three replicate experiments performed in triplicate. Each experiment used keratinocytes derived from a different normal control dog (n = 3) and one of two different XSCID dogs (n = 2). **C** RT-PCR for the CPV-2 spliced transcripts E1∧E2 and reference gene (GAPDH) in non-infected and infected keratinocytes. Results are shown from one experiment performed in triplicate at 2 DPI. Similar results obtained at 4 and 6 days post infection in all replicate experiments. Data is representative of three independent experiments performed in triplicate.

Both normal and XSCID keratinocytes were infected with CPV-2 and the mRNA expression of the proinflammatory cytokines and ISGs was examined at 2, 4, and 6 days post infection. Neither XSCID nor normal control keratinocytes upregulated any proinflammatory cytokines or ISGs at any time point examined (Figure 4A and B). There was a relative decrease in the mRNA expression for TNF-α, IFN-κ, IFI16, IFIT1, MDA5, and RIG-I in the CPV-2 infected keratinocytes when compared to uninfected keratinocytes at 4 days post infection. This relative decrease was similar for both the XSCID and normal keratinocytes.

Confirmation of CPV-2 infection was performed using RT-PCR for the E1∧E2 spliced transcript of CPV-2. Both normal and XSCID keratinocytes revealed the E1∧E2 spliced transcripts at 2, 4 and 6 days post CPV-2 infection. Figure 4C shows the results from a single time point from one representative experiment out of three.

## Discussion

The function, if any, of the γ_c_-dependent cytokine receptors in keratinocytes has not been well explored. While we found that keratinocytes express mRNA for several of the α-subunits of the γ_c_-dependent cytokine receptors, including IL-4Rα, IL-7Rα, and IL-15Rα, there was variable, often faint expression for γ_c_. It is possible that keratinocytes use alternative γ_c_ -independent pathways to signal through these receptors or γ_c_ is upregulated by different stimuli not present in culture conditions. Alternatively, it is possible that keratinocytes increase γ_c_ expression as they differentiate or that culture conditions do not adequately reflect what is occurring *in vivo*. Many non-hematopoietic cells use the type II IL-4R to respond to IL-4 that is not dependent on γ_c_ [24]. The IL-7Rα subunit may be present as a component of the γ_c_ -independent stromal cell-derived lymphopoietin 1 (TSLP) receptor and not part of the γ_c_ -dependent IL-7R [1]. The cytokine IL-15 is often present in a complex with the IL-15Rα subunit, which enables presentation to other cells expressing γ_c_ in complex with the IL-2/15β subunit [25]; thus, expression of the IL-15Rα subunit does not necessarily indicate expression of the signaling receptor. Additionally, IL-15 is most often regulated at the translational level and not at the transcriptional level [26]. The lack of α-subunits for IL-2R, IL-9R, and IL-21R suggests that these cytokine receptors are likely not important in monolayer cultures. Several studies have found that IL-15 and IL-21 do play important roles in keratinocytes [27], [28], [29], [30].

IL-15 has been shown to play a role in antiviral immunity, where it can mediate the release of IFNs and other antiviral cytokines as well as play an essential role in protective immunity to certain viral infections. Despite the role IL-15 has been show to play in protective immunity to certain viral infections and elaboration of antiviral cytokines, we were unable to detect impaired antiviral cytokine or ISG expression in our ligand stimulated γ_c_ -deficient XSCID keratinocytes [13], [14], [15]. Thus, the role of IL-15 and other γ_c_ -dependent cytokines in antiviral immunity within basal keratinocytes does not appear to be due to deficient upregulation of antiviral cytokines or their downstream effectors (ISGs) in response to dsDNA.

Initial infection of basal keratinocytes occurs through small wounds within the skin that expose the basal lamina to papillomavirus [31]. As the virus is taken up by the cell into the cytoplasm, it is released from its capsid and the dsDNA viral genome is transported to the nucleus [31], [32]. It is at this stage that the virus could potentially be recognized by one of the cytosolic dsDNA sensors to trigger upregulation of type I IFNs and ISGs. Additionally, during initial transcription of the early viral genes there is potential for dsRNA intermediates to trigger an antiviral response through the dsRNA sensors. However, this does not seem to be the case for canine keratinocytes upon initial CPV-2 infection. Neither normal nor keratinocytes from XSCID dogs initiated an antiviral response to initial infection with CPV-2. This is in spite of the ability of both XSCID and normal keratinocytes to respond to the dsDNA agonist Poly(dA:dT). It is likely that papillomaviruses have evolved immune escape mechanisms during this initial period of infection in order to avoid immune detection and that these mechanisms are similar between normal and XSCID dogs [33], [34], [35]. The stimulation assays on monolayer cultures reflect what is occurring in the basal and suprabasal keratinocytes, but do not account for terminally differentiated keratinocytes or the presence of other cell types within the epidermis, which is more typical of what is occurring *in vivo*. This suggests that it is not the initial infection of basal keratinocytes that is different between normal and XSCID patients but may be the combined effect of multiple epidermal cell types or the fully differentiated epidermis that effectively creates the antiviral environment required for PV immunity.

As infection progresses, the basal keratinocytes will divide and eventually terminally differentiate through the stratified epidermis. Differentiation of the keratinocyte triggers expression of different viral genes, until eventually the viral genome is replicated and packaged up into virions [31]. At this stage, the viral early and late proteins as well as tumor-associated antigens would be available to be processed and potentially presented to antigen presenting cells. Langerhans cells are important antigen presenting cells within the epidermis that play a role in linking the innate and adaptive immune systems. Lack of γ_c_ function in Langerhans cells or Natural Killer cells, resulting in impaired IL-15R signaling, could be another source for decreased activation of an antiviral response or tumor immunosurveillance. Future investigations into dendritic cell and Langerhans cell function in XSCID dogs, with specific focus on antiviral defense, are warranted.

In conclusion, we found that both normal and XSCID canine keratinocytes responded similarly to poly(dA:dT) by upregulating mRNA expression of antiviral cytokines and ISGs; neither normal nor XSCID keratinocytes upregulated antiviral cytokines in response to infection with CPV-2. These findings suggest that the increased susceptibility to papillomavirus infection in XSCID patients does not seem to be due to an inherent defect within the cytokine response of their γ_c_ -deficient keratinocytes upon initial CPV-2 infection.
